# Image J as the quantification tool in endosonography strain elastography may be reflected in the disturbance of endocrine pancreatic dysfunction

**DOI:** 10.1002/deo2.407

**Published:** 2024-09-22

**Authors:** Ken Nakamura, Seiji Futagami, Shuhei Agawa, Yoshiyuki Watanabe, Tomohide Tanabe, Takeshi Onda, Mayu Habiro, Rie Kawawa, Kumiko Kirita, Nobue Ueki, Katsuhiko Iwakiri

**Affiliations:** ^1^ Division of Gastroenterology Nippon Medical School Tokyo Japan; ^2^ Division of Gastroenterology Kawasaki Rinko General Hospital Kanagawa Japan

**Keywords:** BT‐PABA test, early chronic pancreatitis, ELAST blue, endosonography, HOMA‐β

## Abstract

**Objectives:**

Pancreatic fibrosis is one of the main pathological features of chronic pancreatitis (CP), suggesting a strong relationship between CP and pancreatic ductal cancer. There was no available data about pancreatic fibrosis and pancreatic dysfunction in the early CP (ECP) using endosonography (EUS).

**Methods:**

Asymptomatic patients with pancreatic enzyme abnormalities (AP‐P; *n* = 56) and patients with ECP (*n* = 21) were determined by the absence of abnormal findings on upper gastrointestinal endoscopy, abdominal ultrasonography, and abdominal computed tomography. An Olympus EUS (GF‐UCT 260; Olympus) was used to perform EUS. Open software “Image J”, developed by NIH, was used to measure the surface area fraction of the designated elastic blue region. The maximum value among the pancreatic head, pancreatic body, and pancreatic tail was defined as the ELST‐blue score. The exocrine and endocrine pancreatic functions were evaluated using the *N*‐benzoyl‐l‐tyrosyl‐p‐aminobenzoic acid (BT‐PABA) test and homeostasis model assessment of β‐cell function (HOMA‐β) value, respectively.

**Results:**

EUS score, lobularity, and hyperechoic foci/strands in patients with ECP were significantly (*p* < 0.001) higher than those in patients with AP‐P. In addition, there were no significant differences in the BT‐PABA test (73.1 ± 25.5, 68.5 ± 15.6) and HOMA‐β (93.1 ± 67.4, 73.5 ± 139.7) between patients with ECP and AP‐P. The ELST‐blue score measured by image J as the quantification tool in EUS strain elastography in patients with ECP was significantly higher (*p* = 0.002) than that in patients with AP‐P. Interestingly, the ELST‐blue score was significantly associated with HOMA‐β in patients with ECP.

**Conclusions:**

The ELST‐blue score may be a useful tool for the evaluation of endocrine pancreatic dysfunction in the ECP.

## INTRODUCTION

Chronic pancreatitis (CP) is a progressive condition caused by several factors and characterized by pancreatic fibrosis and dysfunction. However, diagnosis of CP is especially difficult to diagnose at an early stage. In Japan, to prevent the early CP (ECP) from advancing into CP, new strategies for addressing CP in its initial stages have been proposed as ECP.^1^ Early diagnosis and intervention against CP are important for improving the prognosis of CP.

We also reported that the endosonographic score in patients with ECP was significantly higher than that in asymptomatic patients with pancreatic enzyme abnormalities (AP‐P) in small‐sized studies. Recently, sustained inflammation and fibrosis induced by the activation of SREBP‐1 through the serum lipid levels in the pancreas may lead to the progression of pancreatitis via the upregulation of transforming growth factor‐β^2^, ^3^ and may result in the increase of endosonography (EUS) score. Since the treatment for ECP may prevent the progression of CP and the development of pancreatic cancer, the early diagnosis of ECP is critical.

In our data, almost 10% of patients with ECP exhibit severe exocrine pancreatic dysfunction, which increases the risk of pancreatic cancer.^4^ We also reported that the reduction of exocrine pancreatic function related to the risk factor for pancreatic cancer in patients with ECP was worse than that in AP‐P.^4^ Therefore, the evaluation of pancreatic function may be important to distinguish high‐risk groups for pancreatic cancer from patients with CP.

It is mostly difficult to evaluate the progression of pancreatic inflammation and fibrosis using abdominal computed tomography (CT) and abdominal ultrasonography (US) and blood examinations even if the deficiencies of pancreatic function intensely appear. Although we can evaluate pancreatic inflammation and fibrosis using EUS, there was a limitation to the evaluations about the distributions of pancreatic inflammation and fibrosis. Iglesias‐Garcia et al. reported using the strain ratio to diagnose CP using EUS elastography.^5^ They examined the average strain ratios in the head, body, and tail of the pancreas. In addition, there was no available data on the relationship between exocrine and endocrine pancreatic function and pancreatic fibrosis using EUS in patients with ECP.

In this study, we aimed to evaluate the grades of pancreatic fibrosis using Image J for patients with ECP and AP‐P and compared the degree of pancreatic fibrosis with pancreatic function such as exocrine and endocrine pancreatic dysfunction to distinguish the high‐risk group from patients with CP.

## METHODS

### Patients

Seventy‐seven patients with pancreatic enzyme abnormalities underwent upper gastrointestinal (GI) endoscopy, abdominal US, and abdominal CT between April 2019 and October 2022. Patients were recruited from Nippon Medical School Musashi Kosugi Hospital and Nippon Medical School Hospital. AP‐P (*n* = 56) was determined by the absence of abnormal findings on upper GI endoscopy and abdominal US. According to the Japan Pancreatic Association, four clinical criteria, including epigastric pain and the presence of more than two features of EUS, are needed to diagnose ECP (*n* = 21).^6^ Written informed consent was obtained from all patients prior to undergoing upper GI endoscopy, abdominal US, and CT scans to evaluate dyspeptic symptoms. The study protocol was approved by the Ethics Review Committee (620‐3‐15) of Nippon Medical School Hospital.

### Definition of pancreatic enzyme abnormalities

Serum trypsin, PL‐A2, lipase, p‐amylase, and elastase‐1 levels were measured using the same automated chemistry analyzer (AU 5822 analyzer; Beckman Coulter). Pancreatic enzyme abnormalities were defined as the absence of abnormal imaging findings (upper GI endoscopy, abdominal US, or abdominal CT) and any pancreatic enzyme abnormalities, including p‐amylase, lipase, trypsin, PL‐A2, and elastase‐1.

### Endosonographic assessment

An Olympus EUS (GF‐UCT 260; Olympus) was used to perform EUS under conscious sedation on patients with AP‐P and ECP. Endosonographic features were determined based on a previous study.^6^ Patients with ECP were diagnosed according to at least two out of four EUS images and clinical findings of two or more symptoms including repeated attacks of epigastric pain, abnormalities in blood/urine pancreatic enzymes, exocrine pancreatic dysfunction, and a chronic alcohol intake (60 g/day).^6^ EUS score (from 0 to 4) is estimated by the sum of the above EUS findings such as hyperechoic foci or strands, lobularity, hyperechoic MPD margin, and dilated side branches.

### Elasticity of pancreatic tissue using EUS elastography

EUS elastography is an imaging technique that visualizes tissue elasticity using 256 levels of color, with soft tissue appearing red and hard tissue appearing blue.^7^

Elastography measurement methods include strain elastography and shear‐wave elastography. In this study, we evaluated strain elastography using the Olympus GIF‐UCT260 convex endosonographic videoscope and EU‐ME2 ultrasound processor. Strain elastography measures the amount of point variation due to pressurization in real time. Elastography measurement was made from the gastric body for the pancreatic body and tail, and from the duodenal bulb for the pancreatic head, respectively, using an EUS scope. At that time, measurement was taken at one location for several seconds, and an average image was created over several seconds using the device. Using this method, each area was measured twice. The obtained results were analyzed using the following procedure.

### Measurement of pancreatic fibrosis by Image J

**FIGURE 1:**
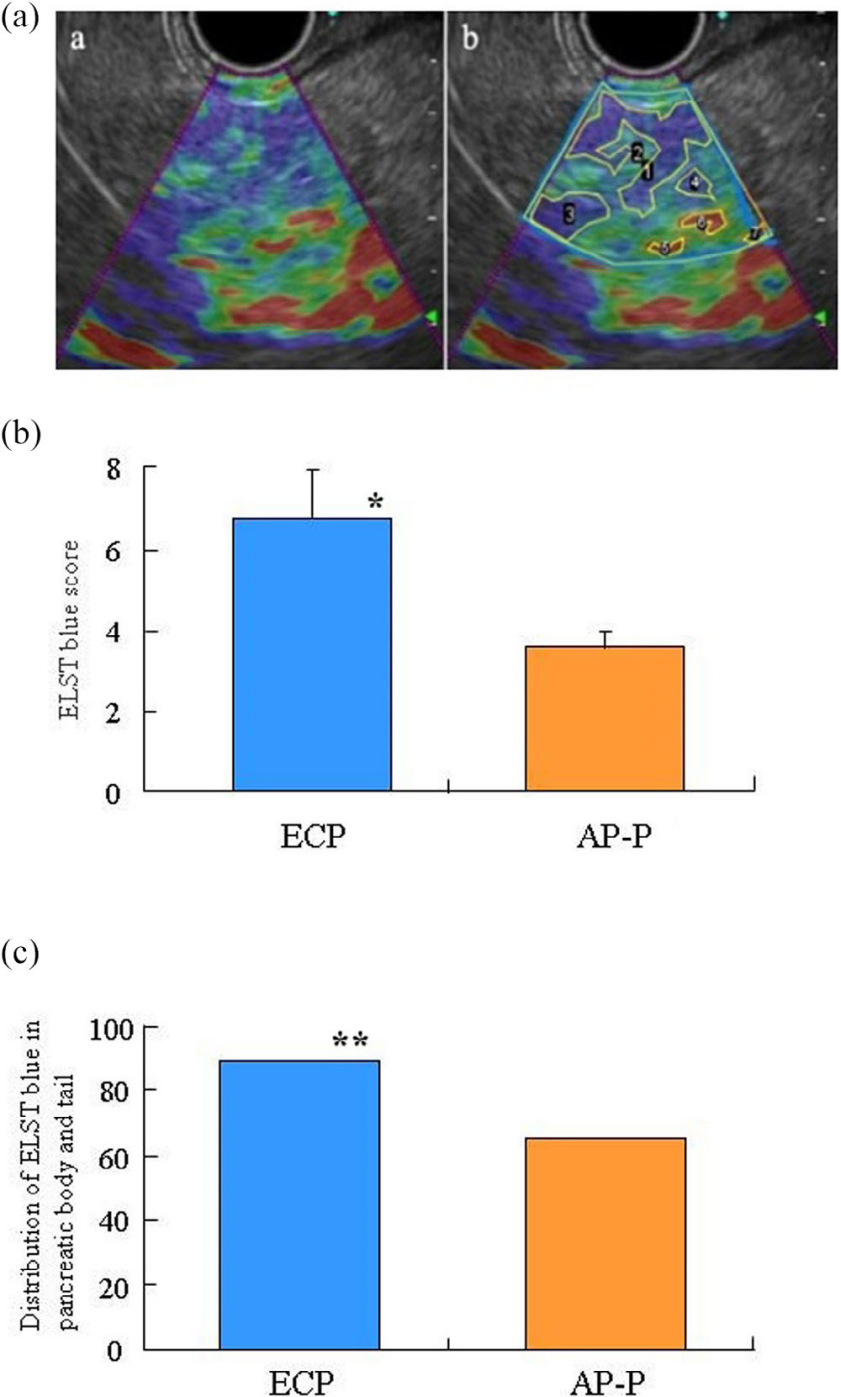
Comparison of ELST‐blue score between patients with early chronic pancreatitis (ECP) and asymptomatic patients with pancreatic enzyme abnormalities (AP‐P). (a) Measurement of Image J. ELST‐blue score = area 1 + 2 + 3+ 4/entire pancreas area. (b) ELST‐blue score in patients with ECP was significantly (*p* = 0.002) higher than that in patients with AP‐P. * versus patients with AP‐P, *p* = 0.002. (c) The distribution of ELST‐blue in the pancreas body or pancreas tail of patients with ECP was significantly higher (*p* = 0.03) than that in patients with AP‐P. ** versus patients with AP‐P, *p* = 0.03.

We analyzed the strain elastography using the open‐source software ‘Image J’ developed by the National Institution of Health. The ratio of the blue area to the entire pancreas was measured in the pancreatic head, body, and tail, respectively. “ELST‐blue” was defined as the maximum value of the mean elastic blue area ratio in three parts in each area (pancreatic head, body, and tail; Figure 1a). Determined ELST‐blue was compared among patients with AP‐P and ECP.

### *N*‐benzoyl‐l‐tyrosyl‐p‐aminobenzoic acid test

The *N*‐benzoyl‐l‐tyrosyl‐p‐aminobenzoic acid (BT‐PABA) test was performed to evaluate exocrine pancreatic function. Exocrine pancreatic insufficiency was diagnosed using a cutoff value of 70%.

### Measurement of homeostasis model assessment of β‐cell function

Homeostasis model assessment of β‐cell function (HOMA‐β) is a method for estimating β‐cell function based on fasting glucose and insulin concentrations.^8^ Fasting blood samples were obtained from participants after an overnight fast. Plasma glucose levels and insulin levels were determined using an automated chemistry analyzer. HOMA‐β was calculated using the following formula.

HOMA‐β = 20 × fasting insulin [µU/mL] / (fasting glucose [mmol/L] – 3.5)

### Statistical analysis

The Mann‐Whitney U‐test and the two‐tailed unpaired *t*‐test were used to compare continuous and categorical variables, respectively, between the groups. The Shapiro‐Wilk test was used to determine whether the data followed a normal distribution. Pearson's correlation coefficient was used for normally distributed data, while Spearman's correlation coefficient was used for non‐normally distributed data. All analyses were performed using SPSS (version 27.0; IBM Corp.), and a *p*‐value less than 0.05 was considered significant.

## RESULTS

### Comparison of clinical characteristics and clinical data between patients with ECP and AP‐P

There were no significant differences in age, gender, BMI, smoking (BI), or alcohol consumption between patients with ECP and AP‐P (Table 1). Moreover, there were no significant differences in the ratios of abnormal pancreatic enzyme abnormalities of p‐amylase, lipase, trypsin, and PLA2 between patients with ECP and AP‐P (Table 2).

**TABLE 1:** Comparison of clinical characteristics between patients with early chronic pancreatitis and asymptomatic patients with pancreatic enzyme abnormalities.

	ECP (*n* = 21)	AP‐P (*n* = 56)	*p‐*value
Age (years)	63.2 ± 3.5	58.4 ± 1.8	0.072
Gender (F/M)	11/10	37 / 19	0.270
BMI (kg/m^2^)	22.5 ± 0.7	21.5 ± 0.5	0.171
Smoking (BI)	203 ± 137	51.3 ± 22.6	0.851
Alcohol consumption (g/day)	7.24 ± 3.38	4.45 ± 1.45	0.282

Abbreviations: AP‐P, asymptomatic patients with pancreatic enzyme abnormalities; BI, Brinkman Index; BMI, body mass index; ECP, early chronic pancreatitis.

Data are mean ± SE.

Statistical analysis of age, BMI, smoking, and alcohol was performed using Mann‐Whitney's U test, and gender was performed using

Pearson's chi‐square test.

**TABLE 2:** Comparison of pancreatic enzyme abnormalities between patients with early chronic pancreatitis and asymptomatic patients with pancreatic enzyme abnormalities.

	ECP (*n* = 21)	AP‐P (*n* = 56)	*p*‐value
P‐amylase (%)	21.1	30.4	0.442
Lipase (%)	23.8	33.9	0.393
Trypsin (%)	38.1	60.7	0.076
PLA2 (%)	23.8	44.6	0.095
Elastase‐1 (%)	6.25	4.35	0.760

Abbreviations: AP‐P, asymptomatic patients with pancreatic enzyme abnormalities; ECP, early chronic pancreatitis; PLA2, phospholipase A2.

Data on pancreatic enzymes represent the percentage of patients with abnormal values.

Statistical analysis for the percentage of patients with abnormal values of pancreatic enzyme was performed using Pearson's chi‐square test.

### Comparison of EUS score and EUS features between patients with ECP and AP‐P

EUS score in patients with ECP was significantly (*p* < 0.001) higher than that in patients with AP‐P (Table 3). Although lobularity and hyperechoic foci/strands in ECP were significantly (*p* < 0.001 and *p* < 0.001, respectively) higher compared to those in patients with AP‐P, there were no significant differences in hyperechoic MPD margin and diluted side branches between patients with ECP and AP‐P (Table 3).

**TABLE 3:** Comparison of endosonography (EUS) score and EUS features between patients with early chronic pancreatitis (ECP) and asymptomatic patients with pancreatic enzyme abnormalities (AP‐P).

	ECP (*n* = 21)	AP‐P (*n* = 56)	*p*‐value
EUS score	1.71 ± 0.16	0.52 ± 0.07	< 0.001
Lobularity	0.48 ± 0.11	0.05 ± 0.03	< 0.001
Hyperechoic foci/Strands	0.67 ± 0.11	0.20 ± 0.05	< 0.001
Hyperechoic MPD margin	0.43 ± 0.11	0.23 ± 0.06	0.091
Dilated side branches	0.14 ± 0.08	0.04 ± 0.03	0.091

Abbreviations: AP‐P, asymptomatic patients with pancreatic enzyme abnormalities; ECP, early chronic pancreatitis; EUS, endosonography; MPD, main pancreatic duct.

Data are mean ± SE.

Statistical analysis of the EUS score was performed using Mann‐Whitney's U test.

### Comparison of exocrine and endocrine pancreatic dysfunction between patients with ECP and AP‐P

Since there was no available data about pancreatic function in patients with ECP, we compared exocrine and endocrine pancreatic function between patients with ECP and AP‐P. To compare exocrine and endocrine pancreatic dysfunction between patients with AP‐P and ECP, we measured the BT‐PABA test and HOMA‐β value in these patients. Then, there were no significant differences in the BT‐PABA test (70.6 ± 6.0, 66.0 ± 2.6) and HOMA‐β (105 ± 16, 101 ± 19) between patients with ECP and AP‐P (Table 4).

**TABLE 4:** Comparison of exocrine and endocrine pancreatic dysfunction between patients with early chronic pancreatitis (ECP) and asymptomatic patients with pancreatic enzyme abnormalities (AP‐P).

	ECP (n = 21)	AP‐P (n = 56)	*p*‐value
BT‐PABA (%)	70.6 ± 6.0	66.0 ± 2.6	0.366
HOMA‐β (%)	105 ± 16	101 ± 19	0.774

Abbreviations: AP‐P, asymptomatic patients with pancreatic enzyme abnormalities; BT‐PABA, *N*‐benzoyl‐L‐tyrosyl‐p‐aminobenzoic acid test; ECP, early chronic pancreatitis; HOMA‐β, homeostatic model assessment of beta cell function.

Data are mean ± SE.

Statistical analysis of exocrine and endocrine dysfunction was performed using Mann‐Whitney's U test.

### Comparison of ELST‐blue score between patients with ECP and AP‐P

To compare the grade of pancreatic fibrosis between patients with ECP and AP‐P, we measured the ELST‐blue score in two groups using EUS. ELST‐blue score in patients with ECP was significantly higher (*p* = 0.002) than that in patients with AP‐P (Figure 1b). We compared whether there were any differences in the distribution of ELST‐blue among the pancreas head, pancreas body, and pancreas tail. The distribution of ELAST‐blue in the pancreas body or pancreas tail of patients with ECP was significantly higher (*p* = 0.03) than that in patients with AP‐P (Figure 1c).

### Relationship between ELST‐blue score and HOMA‐β and BT‐PABA test in patients with ECP

**FIGURE 2:**
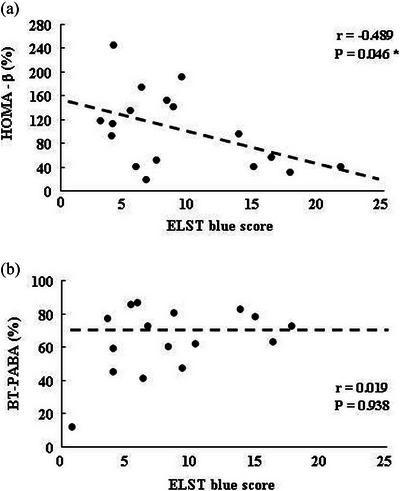
Relationship between ELST‐blue score and homeostatic model assessment of beta cell function (HOMA‐β) in patients with early chronic pancreatitis (ECP). ELST‐blue score was significantly (*p* = 0.046) associated with HOMA‐β in patients with ECP.

ELST‐blue score was significantly (p = 0.046) associated with HOMA‐β in patients with ECP (Figure 2a), albeit there was no significant relationship between the BT‐PABA test and ELST‐blue score in patients with ECP (Figure 2b). In contrast, in patients with AP‐P, there were no significant associations between the ELST‐blue score, BT‐PABA test, and HOMA‐β (data not shown).

### Relationship between EUS score and BT‐PABA test and HOMA‐β in patients with ECP

There was no significant relationship (*p* = 0.872) between the EUS score and BT‐PABA test in patients with ECP. In addition, there was also no significant relationship (*p* = 0.285) between EUS score and HOMA‐β in patients with ECP.

### Multiple logistic regression for ELST‐blue score

To clarify which factors affect ELST‐blue score, we performed multiple logistic regression for ELST‐blue score. However, various factors such as age, smoking, alcohol consumption, trypsin level, T‐PABA test, HOMA‐β, and HbA1c value were not significant (*p* = 0.677, 0.500, 0.375, 0.609, 0.077, 0.689, and 0.229) associated with ELST‐blue score.

## DISCUSSION

Major findings in this study were as follows: 1) ELST‐blue score measured by image J as the quantification tool in EUS strain elastography in patients with ECP was significantly higher (*p* = 0.002) than that in patients with AP‐P., and 2) there was a negatively significant relationship between ELAST blue score and HOMA‐β value in patients with ECP.

EUS is also superior to non‐invasive imaging tools in diagnosing parenchymal and ductal changes, especially during the early stage of the disease. Therefore, when CT and MRI show negative results in patients who are suspected of having CP, EUS should be performed.^9^ We could not detect any findings in abdominal CT and US, however, there were significant differences in total EUS score and lobularity and hyperechoic foci/ strands between patients with ECP and AP‐P. Therefore, we could effectively distinguish patients with ECP from AP‐P using EUS findings. Then, pancreatic fibrosis is one of the main pathological features of CP, suggesting a strong relationship between CP and pancreatic ductal cancer (PDAC). However, there was no available data about pancreatic fibrosis and pancreatic dysfunction in the ECP using EUS. Therefore, we tried to evaluate the grade of pancreatic fibrosis using the ELST‐blue score measured by image J as the quantification tool in EUS strain elastography in patients with ECP. Interestingly, the ELST‐blue score in patients with ECP was significantly higher than that in patients with AP‐P. Therefore, the ELST‐blue score may be a useful tool to evaluate the disturbance of endocrine pancreatic function in patients with ECP.

Whitcomb et al. have reported that “mechanistic definition” may be the critical pathway to CP and pancreatic cancer.^10^ In contrast, it is very difficult to find early‐stage pancreatic cancer using several examinations such as abdominal US, abdominal CT, and MRCP. Therefore, we tried to determine whether the clinical characteristics and exocrine and endocrine pancreatic function of patients with ECP may be different from those of patients with AP‐P and we would like to find which factors connect to the promotion of CP and pancreatic cancer. Thus, there was no available data about exocrine and endocrine pancreatic functions in patients with ECP and AP‐P. In our data, there were no significant differences in exocrine and endocrine pancreatic functions between patients with ECP and AP‐P as described in Table 4. Although pancreatic inflammation and fibrosis in patients will be considered to advance accompanied by the disturbances of exocrine and endocrine pancreatic function, there was no significant relationship between EUS score and pancreatic function such as BT‐PABA test and HOMA‐β. EUS features such as lobularity with honey‐combing, hyperechoic stranding, and hyperechoic MPD margin may be reflected in various fibrosis including interlobular fibrosis, bridging fibrosis, and periductal fibrosis.^11^, ^12^, ^13^ Further studies will be needed to clarify why the EUS score was not associated with the reduction of exocrine and endocrine pancreatic function. Then, we tried to determine whether the ELST‐blue score was negatively associated with HOMA‐β as an endocrine function. Although pancreatic inflammation causes fibrosis of the pancreas, inflammation of the pancreas is not uniformly extended throughout the pancreas. Actually, patients with ECP exhibit localized inflammation and fibrosis detected by EUS. Localized and sustained inflammation in the pancreatic head or pancreatic tail was mostly found in patients with ECP. In contrast, pancreatic inflammation was uniformly observed in patients with CP. Therefore, it is the reason why the ELST‐blue score was defined using the maximum value among the three pancreatic regions in patients with ECP. We can find a significant relationship between the ELST‐blue score and the HOMA‐β value. ELST‐blue score which indicated the area of pancreatic fibrosis may be reflected in the reduction of endocrine pancreatic function, however, the ELST‐blue score was not linked with the BT‐PABA test as exocrine pancreatic function. Previous studies have reported that sustained inflammation‐fibrosis accompanying the disturbance of exocrine and endocrine tissues was associated with a significant loss of β cells and hence a progressive reduction in endocrine function.^14^, ^15^ In our data, in ECP, the ELST‐blue score which reflected on pancreatic fibrosis may be associated with the reduction of β cells. After the progression of CP, ELST‐blue score may be associated with exocrine pancreatic function as well as endocrine pancreatic function.

Pancreatic fibrosis is one of the main pathological features of CP, suggesting a strong relationship between CP and PDAC.^16^ Pancreatic fibrosis is a defining hallmark of PDAC occurrence and prognosis. A set of genes was recently reported to be involved in the development of pancreatic fibrosis and PC. CXCR2 knockout mice showed higher levels of pancreatic fibrosis and increased the malignancy of PDAC in vivo, indicating that CXCR2 played an important role in the transition from pancreatic fibrosis to PC.^17^ Therefore, it may be critical to evaluate pancreatic fibrosis in ECP using EUS. Thus, the EUS score is useful to distinguish patients with ECP from patients with AP‐P. In contrast, the ELST‐blue score which reflected on pancreatic fibrosis may be available to evaluate endocrine pancreatic function in ECP. However, we could not find which factors contribute to the ELST‐blue score using multiple logistic regression analysis. Further studies will be needed to clarify which factors affect to ELST‐blue score in more numbers of cases.

Taken together, the limitations of this study are single‐center study and small‐sized study. In addition, further study will be needed to clarify whether the ELST‐blue score was associated with endocrine and exocrine pancreatic function in patients with advanced CP and whether the subgroup with a high ELST‐blue score in ECP may advance to CP in follow‐up.

## CONFLICT OF INTEREST STATEMENT

None.

## ETHICS STATEMENT

The study protocol was approved by the Ethics Review Committee (620‐3‐15) of Nippon Medical School Hospital. Written informed consent was obtained from all patients prior to undergoing upper GI endoscopy, abdominal US, and CT scans to evaluate dyspeptic symptoms.
